# *GLCCI1* rs37973: a potential genetic predictor of therapeutic response to inhaled corticosteroids in Chinese chronic obstructive pulmonary disease patients

**DOI:** 10.1038/srep42552

**Published:** 2017-02-10

**Authors:** Yuan Lei, Yiping Gao, Jinkun Chen, Miao Li, Xiaomei Wu, Qin Ning, Jianping Zhao, Weining Xiong, Yongjian Xu, Jungang Xie

**Affiliations:** 1 Department of Respiratory and Critical Care Medicine, National Clinical Research Center of Respiratory Disease, Tongji Hospital, Tongji Medical College, Huazhong University of Science and Technology, 1095 Jiefang Avenue, Wuhan, 430030, China; 2 Acadia Junior High School, Winnipeg, Manitoba, R3T 3B3, Canada; 3 Department of Infectious Disease, Institute of Infectious Disease, Tongji Hospital, Tongji Medical College, Huazhong University of Science and Technology, 1095 Jiefang Avenue, Wuhan, 430030, China

## Abstract

Inhaled corticosteroids (ICSs) are widely prescribed in chronic obstructive pulmonary disease (COPD). However, little is known about predictors of ICSs therapeutic response. To investigate whether the variation in glucocorticoid-induced transcript 1 (*GLCCI1*) rs37973 is associated with ICS efficacy. A total of 204 clinically stable COPD patients were recruited and administered to inhaled fluticasone propionate/salmeterol combination (500/50 ug, twice daily) for 24 weeks. We genotyped the functional rs37973 and mainly assessed its effects on changes in lung function. *In vitro*, neutrophils isolated from parts of patients were incubated with various concentrations of dexamethasone (0, 10^−6^ M and 10^−4^ M) in the presence or absence of cigarette smoke extract, apoptosis was then assessed by flow cytometry. Patients with the homozygous GG genotype (increases of 15.3 ± 33.2 mL) had significantly poorer improvement in FEV_1_ than those with the AA (92.7 ± 29.6 mL; *p* < 0.001) or AG (59.4 ± 26.9 mL; *p* < 0.001) genotypes after 24-week treatment. *In vitro*, dexamethasone had less inhibitory effect of neutrophil apoptosis on GG genotype, which further validated the presence of mutant allele ‘G’ might negatively affect glucocorticoid responsiveness irrespective of smoking status. The GG genotype of rs37973 may associated with decreased ICSs efficacy in Chinese COPD patients.

Chronic obstructive pulmonary disease (COPD) is a leading and increasing cause of mortality worldwide^1^, which lead to persistent airflow limitation, airway remodeling and progressive deterioration in lung function. Cigarette smoking is one of the major risk factors for the ongoing inflammation in airways^2^, and inhaled corticosteroids (ICSs) are the mainstay of anti-inflammatory therapy. Current guidelines recommend the use of ICSs combining with long-acting *β*_*2*_-agonists for COPD patients at high risk of exacerbations^3^. However, the efficacy shows high inter-individual variability with numerous patients having insufficient response. And prolonged ICSs therapy carries the risk of local and systemic side effects such as oropharyngeal candidiasis, hoarseness, pneumonia^4^,^5^, osteoporosis etc^6^. Therefore, identification of patients who are prone to nonresponse is becoming increasingly important.

Recently a novel pharmacogenetic variation in the glucocorticoid-induced transcript 1 gene (*GLCCI1*) has been studied intensively in asthma^7^,^8^,^9^,^10^,^11^, since Tantisira and colleagues revealed in 2011 that the functional rs37973 polymorphism, which was in complete linkage disequilibrium with rs37972, might substantially cause a decreased response to ICSs therapy in non-Hispanic white asthmatics^12^. Subsequently, Van den Berge *et al*. further investigated the effect of *GLCCI1* variant rs37972 on glucocorticoid responsiveness in 63 COPD patients. And then extended the findings of Tantisira *et al*. from asthma to COPD, by showing that *GLCCI1* was also associated with changes in pulmonary function after ICS therapy in COPD^13^. However, due to small numbers of participants and lack of functional validation, the precise role of *GLCCI1* in COPD was far from definable.

Neutrophilic inflammation is a prominent feature of COPD in airways as well as circulation. This persistent and abnormal inflammation is highly correlate with disease progression, and apoptosis is a crucial resolution for non-phlogistic clearance of neutrophils^14^. However, glucocorticoids delay neutrophil apoptosis *in vitro* cell culture. That may partly account for relative less effectiveness of ICSs in COPD than asthma^15^. Located in *GLCCI1* promoter region, the mutant allele ‘G’ of rs37973 was confirmed to down-regulate the expression of *GLCCI1 in vitro* functional analysis^12^. *GLCCI1* induction was an early maker of apoptosis in glucocorticoid-treated thymoma cells^16^. Hence, we postulated that neutrophils, isolated from COPD patients with different genotypes of rs37973, might also responded differently to glucocorticoids stimulation *in vitro*.

Unlike asthma, although widely prescribed, very few pharmacogenetics studies in COPD have focused on the role of genetic variants in ICS therapeutic response^17^,^18^. Based on their findings, we aimed to further investigate whether the functional rs37973 variant is associated with long-term ICS therapeutic response in Chinese COPD patients. Moreover, we aimed to verify the potential predictive value of rs37973 variant *in vitro* cell study of dexamethasone-mediated neutrophil apoptosis.

## Results

### Participants

A total of 209 clinically stable COPD patients were recruited, among them, 204 eligible patients were finally included in our study (4 lost to follow-up and 1 migrated). The demographic characteristics and lung function at baseline were homogeneous between groups stratified by the rs37973 genotype (Table 1). The mean age was 67.0 years old. A total of 79.4% patients were men and 53.4% were current smokers with a history of smoking more than 20 pack-years. The mean post-bronchodilator forced expiratory volume in one second (FEV_1_) was 1.22 liters, which was 46.31% of the predicted value. All of our patients were in category C or D according to the Global Initiative for Chronic Obstructive Lung Disease (GOLD) criteria^3^, which meant high risk of COPD exacerbation.

### Genotype and allele frequencies

About the rs37973 genotype, 59 (28.9%) individuals were homozygous for the major ‘A’ allele, 50 (24.5%) individuals were homozygous for the mutant ‘G’ allele and 95 (46.6%) were AG heterozygotes. The minor allele ‘G’ frequency was 0.478 and all genotype frequencies were in Hardy-Weinberg equilibrium (*p* = 0.340).

### Association between rs37973 genotype and lung function changes

**Figure 1:**
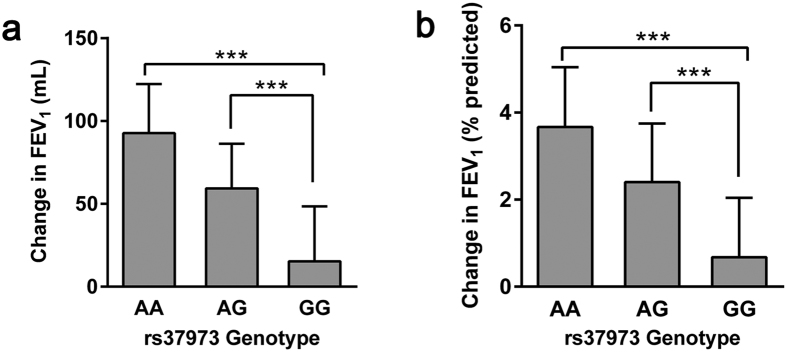
Changes in lung function after 24-week treatment with ICS according to *GLCCI1* rs37973 genotype. The GG genotype was significantly associated with poor improvement in **(a)** FEV_1_ and **(b)** FEV_1_ % of predicted, relative to AA and AG genotypes. Data were represented as mean ± SD. ^***^P < 0.001.

**Figure 2:**
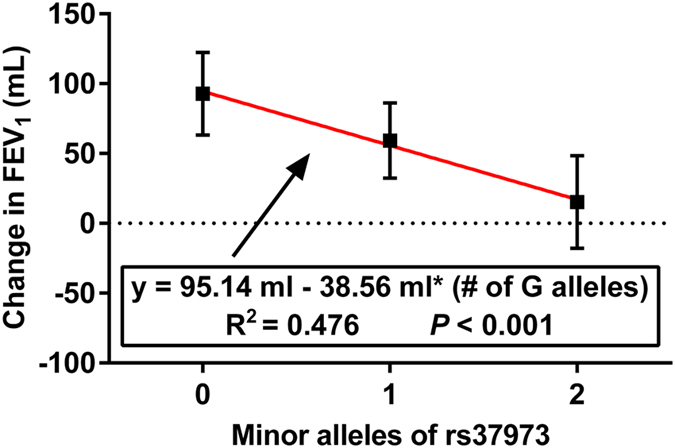
Effect of per minor allele ‘G’ of rs37973 (coded as 0, 1, or 2) on changes in FEV_1_. The equation of fitted linear regression suggested that each additional copy of ‘G’ allele corresponded to a lower ICS efficacy.

After 24-week treatment of ICS, patients with the homozygous GG genotype (increases of 15.3 ± 33.2 mL) had significantly poorer improvement in FEV_1_ than those with the AA (92.7 ± 29.6 mL; *p* < 0.001) or AG (59.4 ± 26.9 mL; *p* < 0.001) genotypes (Fig. 1a). As for FEV_1_ % of predicted, the GG homozygotes also increased remarkably lower (0.68 ± 1.36%), compared with the AA homozygotes (3.67 ± 1.38%; *p* < 0.001) or AG heterozygotes (2.40 ± 1.35%; *p* < 0.001), while the overall mean improvement was 2.33 ± 1.73% (Fig. 1b). In addition, the effect of per mutant allele ‘G’ of rs37973 was estimated by regression analysis (Fig. 2), suggesting that each additional copy of ‘G’ allele corresponded to a lower ICS efficacy (R^2^ = 0.476, F = 179.23, *P* < 0.001).

### Effects of smoking status on ICS efficacy

**Figure 3:**
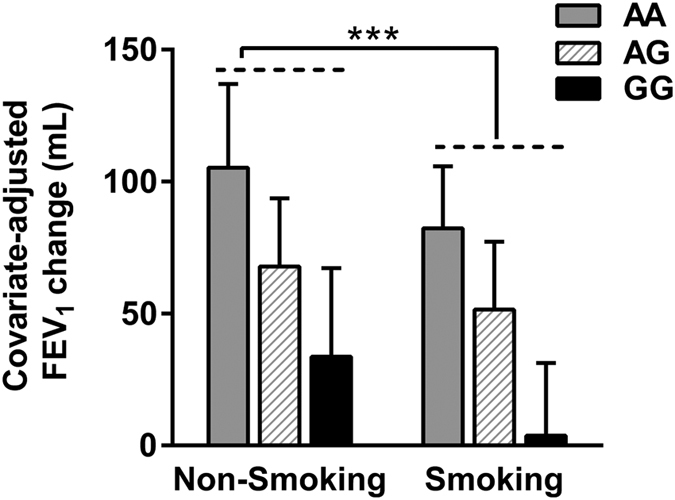
Effects of smoking status and rs37973 genotype on ICS efficacy with adjustment for age, sex and baseline percentage of predicted FEV_1_. Smokers had significant poor mean (±SD) improvement in covariate-adjusted FEV_1_, compared with non-smoking patients after 24-week treatment with ICS. ^***^P < 0.001.

Consistent with previous studies^19^,^20^, we found that smoking status was significant predictor of changes in FEV_1_ (Fig. 3). Smokers had significant poor improvement compared with non-smoking patients (46.8 ± 39.0 mL versus 71.0 ± 38.3 mL; *p* < 0.001), after adjusting for age, sex and baseline percentage of predicted FEV_1_. As for changes in FEV_1_ % of predicted, similar results were observed in smoking and non-smoking patients (unadjusted for covariates, 1.86 ± 1.61% versus 2.89 ± 1.72%; *p* < 0.001).

### Effects of rs37973 genotype on dexamethasone-mediated neutrophil apoptosis

**Figure 4:**
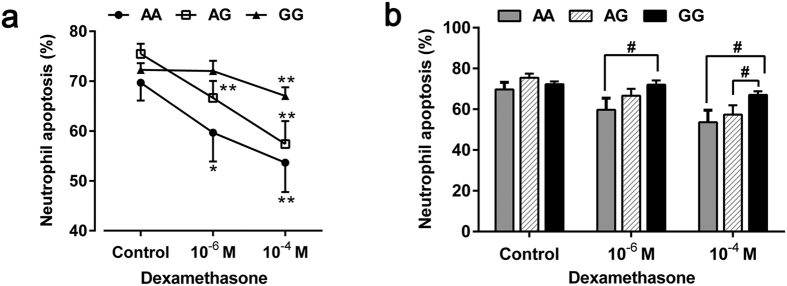
Effects of dexamethasone and rs37973 genotype on neutrophil apoptosis. **(a)** Dexamethasone significantly inhibited neutrophil apoptosis irrespective of genotype in a concentration-dependent manner. **(b)** Dexamethasone had less inhibitory effect of neutrophil apoptosis on GG genotype, relative to AA and AG genotypes. Data were presented as mean ± SEM; ^*^P < 0.05, ^**^P < 0.01 (compared with respective solvent control); ^#^*P* < 0.05.

A total of 43 voluntary patients provided an extra 8 ml venous blood for neutrophil culture *in vitro*, among them, the numbers of AA, AG and GG genotypes of rs37973 were 11, 14 and 18, respectively. Dexamethasone significantly inhibited spontaneous neutrophil apoptosis irrespective of genotype in a concentration-dependent (0, 10^−6^ M and 10^−4^ M) manner under standard culture conditions for 18 h (Fig. 4a). Interestingly, we found that neutrophils isolated from patients with GG genotype didn’t show corresponding reductions in apoptosis at 10^−6^ M, compared with solvent control (the percentage of apoptotic cells: 72.03 ± 2.06% versus 72.27 ± 1.33%; *p* = 0.903).

Previous studies have reported that dexamethasone caused a concentration-dependent inhibition of neutrophil apoptosis, usually evident at 10^−8^ M and maximal at 10^−6^ M of clinically relevant drug concentrations^21^,^22^,^23^. Similar anti-apoptotic effects were also confirmed in AA and AG genotypes of rs37973 in our study. However, since dexamethasone at 10^−6^ M had little effect on GG genotype, we extended our experimental concentration to 10^−4^ M, higher than clinically relevant drug concentrations. Only then did we observed an expected suppression of neutrophil apoptosis by dexamethasone in GG genotype (67.03 ± 1.74% versus 72.27 ± 1.33%; *p* = 0.004). In addition, we observed that neutrophils isolated from patients with AA genotype had significantly decreased percentage of apoptosis compared with those of GG genotype in the presence of both 10^−6^ M (59.68 ± 5.80% versus 72.03 ± 2.06%; *p* = 0.025) and 10^−4^ M (53.64 ± 5.88% versus 67.03 ± 1.74%; *p* = 0.013) dexamethasone, suggesting that dexamethasone had less inhibitory effect on GG genotype (Fig. 4b).

### Dexamethasone attenuated CSE-induced neutrophil apoptosis

**Figure 5:**
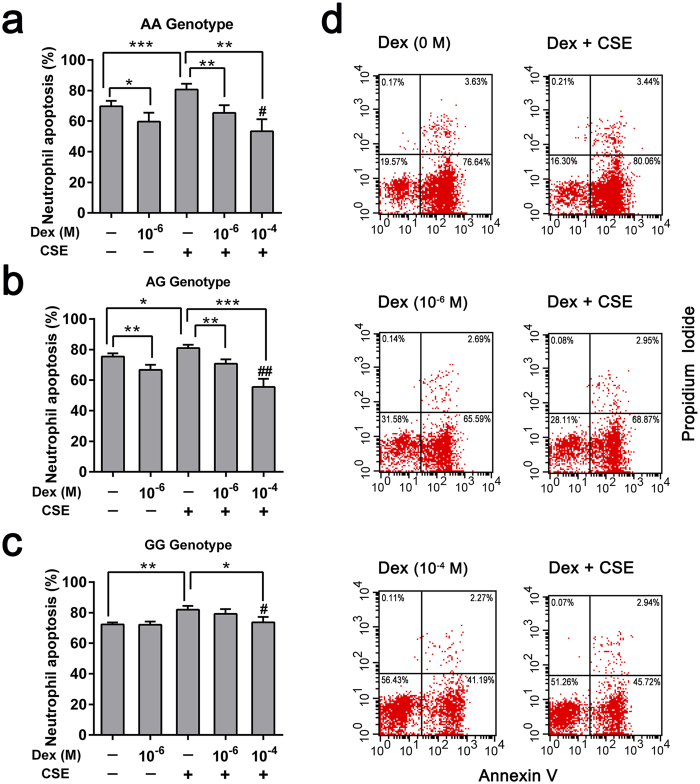
Dexamethasone attenuated CSE-induced neutrophil apoptosis in different genotypes. Neutrophils isolated from COPD patients with the **(a)** AA genotype, **(b)** AG genotype or **(c)** GG genotype were incubated in the presence (+) or absence (−) of Dex or CSE (10 ug/ml) for 18 h. The percentage of neutrophil apoptosis (Ann V ^+^ /PI^−^) was assessed by flow cytometry. **(d)** Representative FACS plots of Ann V/PI staining in one sample cultured with various concentrations of Dex (0, 10^−6^ M and 10^−4^ M), with or without CSE. Data were presented as mean ± SEM. ^*^P < 0.05, ^**^P < 0.01, ^***^P < 0.001; ^#^*P* < 0.05, ^##^*P* < 0.01 (compared with 10^−6^ M Dex- and CSE-treated cells). Dex, Dexamethasone; CSE, cigarette smoke extract.

Cigarette smoke extract (CSE), used in isolated neutrophils system as the underlying inflammatory milieu, could significantly promote neutrophil apoptosis comparing with sham-treated cells of all genotypes. For AA and AG genotypes, dexamethasone reduced the pro-apoptotic effect of CSE in a concentration-dependent manner (10^−6^ M and 10^−4^ M), compared with merely CSE-treated cells (Fig. 5a,b,d). However, we didn’t observe the same effect of dexamethasone on GG genotype at a concentration of 10^−6^ M (Fig. 5c), which was similar to culturing without CSE milieu. CSE-induced neutrophil apoptosis could only be slightly attenuated by a relatively higher concentration (10^−4^ M) of dexamethasone (73.63 ± 3.58% versus 81.94 ± 2.45%; *p* = 0.032).

## Discussion

In our study, we confirmed that *GLCCI1* rs37973 was an important determinant of decreased ICSs therapeutic response in COPD. Patients with homozygous mutant ‘G’ genotype had significantly poor improvement in lung function after 24 weeks of ICS treatment. Moreover, we validated the potential predictive value of rs37973 variant on corticosteroid responsiveness *in-vitro* experiments, showing that dexamethasone had less anti-apoptotic effects on neutrophils isolated from patients with the GG genotype.

Following up the findings of Tantisira *et al*. in asthma^12^, Van den Berge and colleagues firstly investigated the role of *GLCCI1* rs37972 polymorphism in ICS therapeutic response in 63 Dutch COPD patients. After 3 and 30 months treatment of fluticasone with or without added salmeterol, the major allele homozygotes had remarkably greater improvement in FEV_1_ than those with the homozygous mutant genotypes, indicating that *GLCCI1* was also associated with ICS responsiveness in COPD^13^. On the basis of Van den Berge *et al*., a larger sample set, 402 non-Hispanic white COPD patients drawn from two GSK-sponsored clinical studies, were treated with fluticasone furoate monotherapy for 12 weeks. However the results were contrary, showing that the *GLCCI1* rs37973 polymorphism, which was highly correlated with rs37972, didn’t have an effect on FEV_1_ response^24^. Considering the small sample size of Van den Berge *et al*.’s research, the initial association might be a false positive. However, on the other side, ICS monotherapy is not recommended by guidelines^3^, for it is less effective than combination with long-acting *β*_*2*_-agonists. The subjects in GSK study had a lower baseline FEV1 % of predicted and received exclusively ICS monotherapy for only 12 weeks, which might lead to a relative false negative. In addition, the controversial conclusion might also partly result from different ethnics. To better elucidate the possible role of *GLCCI1* in COPD, our research then selected a well-characterized population of 204 Chinese clinical stable COPD patients, and all the patients received inhaled fluticasone propionate added salmeterol therapy for 24 weeks. Combining with *in-vitro* cell functional validated, our findings further confirmed that the functional rs37973 polymorphism held the potential to be a novel genetic predictor of ICS therapeutic response in Chinese COPD patients.

Consistent with previous researches in asthma, the functional rs37973 variant in *GLCCI1* was first identified to cause substantial decrements in ICS therapeutic response in 935 white non-Hispanic adults and children by Tantisira *et al*.^12^, and subsequently validated in 402 European asthmatic children^9^ and 224 Japanese adult patients^7^. However, the role of rs37973 has recently been challenged by a lager well-designed replication study in 7 GSK-sponsored clinical trials including 1924 non-Hispanic white asthmatics, by showing that rs37973 didn’t significantly influence changes in FEV_1_ after 8 or 12 weeks of ICSs treatment^8^. Possible explanations for this discrepant evidence might be divergent end points, different treatment durations and ethnic variations. Both COPD and asthma are chronic airway inflammation characterized by airflow limitation and probably share a common genetic background according to Dutch hypothesis^25^. Notwithstanding the similarities, patients with COPD are generally less responsive to corticosteroids than asthmatics, which may partly result from the distinct difference in key inflammatory cells involved in COPD (neutrophils, macrophages, CD8 + lymphocytes)^26^ and asthma (eosinophils, CD4 + lymphocytes)^27^. As previous studies have shown that glucocorticoids inhibit neutrophil apoptosis while promote eosinophil apoptosis at clinically relevant drug concentrations, usually between 10^−10^ M and 10^−6^ M *in vitro* cell culture^23^,^28^,^29^. Though pharmacogenetics may display a class effect in various disease, considering the differences between COPD and asthma, the effect of rs37973 variant on corticosteroid responsiveness should be interpreted with caution and further prospective validations will be required in both COPD and asthma.

To the best of our knowledge, only one other genetic variant, rs242941 in the corticotrophin releasing hormone receptor 1 (*CRHR1*) had been reported as a probable determinant of corticosteroid responsiveness in COPD^18^. However, the study of Kim *et al*. was small with only 12-week follow-up observation in a total of 87 patients and due to lack of further validation, the role of *CRHR1* in COPD was still indefinable. Although ICSs are widely or even over prescribed^30^, only very few studies demonstrated markers that might associated with corticosteroid responsiveness in COPD. And most of them are limited to short-term response or uncertain clinical features such as bronchodilator responsiveness^31^, frequent exacerbation^32^, sputum eosinophilia etc^33^. No predictors of long-term responsiveness has been established. Thus our research herein highlights a promising new approach of pharmacogenetics to predict long-term ICSs therapeutic efficacy in COPD.

Cigarette smoking is not only the most important cause of COPD, but also associated with accelerated pulmonary function decline and decreased corticosteroid responsiveness^2^,^34^. Our research confirmed those of previous studies by showing that smokers had significant poor improvement in FEV_1_ after 24-week ICS therapy. Therefore, smoking cessation is an essential therapeutic strategy for COPD.

*In vitro* functional validation, consistent with previous studies^21^,^22^,^23^, we observed that dexamethasone inhibited spontaneous neutrophil apoptosis in a concentration-dependent manner under standard culture conditions. Interestingly, in the presence of 10^−6^ M dexamethasone, which was thought to be the maximal inhibitory concentration^22^, neutrophils isolated from patients with the GG genotype of rs37973 didn’t show corresponding reductions in apoptosis with or without CSE milieu. Only when we extended our experimental concentration to 10^−4^ M, did we observed an expected inhibitory effort. In keeping with our clinical related findings, those results further validated the negative effect of mutant allele ‘G’ on corticosteroid responsiveness irrespective of smoking status.

Since apoptosis helps eliminate granulocytes without releasing histotoxic mediators and limit tissue injury, to some extent, an inappropriate delay in apoptosis may be detrimental to control neutrophilic inflammation. Thus, inhibition of neutrophil apoptosis didn’t result in concomitant better clinical response, our experiments could only verify neutrophils with GG genotype responded less to glucocorticoids *in vitro*, neutrophil apoptosis couldn’t act as an indicator of changes in lung function after ICS therapy. However, Marwick *et al*. reported that under severe hypoxic conditions, glucocorticoids lost their ability to promote neutrophil survival and could even reduce the pro-survival effect of inflammatory mediators like GM-CSF, suggesting that glucocorticoids might not account for augmenting neutrophil survival at sites of inflamed tissues^21^. As potent but nonspecific anti-inflammatory drugs, glucocorticoids in COPD are more likely to exert their anti-inflammatory effects through inhibiting lymphocytic inflammation^35^, increasing epithelial barrier function, decreasing oxidative stress etc^36^. The immunopathogenesis of COPD is quite intricate with various inflammatory cells, cytokines and their interactions in different surrounding environment^37^, thus further research imitating *in-vivo* inflammatory environments is clearly needed to investigate the exact anti-inflammatory mechanisms of glucocorticoids in COPD.

However, there are certain limitations in our study. We recruited a selective group of homogeneous COPD patients in a Han Chinese population. Since genetic distribution varies between ethnicities, our results may not generalize to other ethnic groups. Additionally, we only investigated one single potential genetic predictor, which was far from accurately predict ICSs efficacy in COPD. As McGeachie *et al*. reported that even combination of just two genetic variants, *GLCCI1* and *CRHR1*, could achieve better accuracy in predicting ICSs therapeutic response of asthmatics^10^. Although little is known about predictors of corticosteroid responsiveness in COPD, our study herein shed new light on individualized treatment for COPD, indicating that pharmacogenetic may become a promising new approach to predict long-term ICSs efficacy.

Another limitation is that combination therapy with ICS and long-acting *β*_*2*_-agonists (fluticasone propionate/salmeterol) was used in our study, ICS therapeutic response might be influenced by the *β*_*2*_-agonists in some degree. The exact effect of long-acting *β*_*2*_-agonists on ICS is uncertain, but they are believed to have a synergistic interaction^38^. Indeed, COPD patients benefit better from combination therapy than ICS monotherapy, and it’s widely used in practice as recommended by guidelines^3^. But then, we further verified the effect of rs37973 variant on corticosteroid responsiveness *in vitro* cell study. Despite these limitations, we are confident that our principal findings are reliable since the clinical trial and cell experiments consistently indicated the presence of mutant allele ‘G’ might negatively affect corticosteroid responsiveness irrespective of smoking status.

In conclusion, our study reveals that the GG genotype of rs37973 is associated with decreased ICSs efficacy in clinically stable COPD patients in a Han Chinese population.

## Material and Methods

### Participants

From April 2012 through December 2013, a total of 209 outpatients with clinically stable COPD were recruited from Tongji Hospital, Wuhan, China. All the patients were in category C or D according to the GOLD criteria^3^, which defined as a post-bronchodilator FEV_1_/forced vital capacity ratio less than 70%, FEV_1_ less than 50% of the predicted value and/or at least one documented COPD exacerbation in the preceding year before screening. In addition, eligible patients were at least 40 years old. Main exclusion criteria were asthma or respiratory tract infections, receipt of systemic or inhaled corticosteroids within 4 weeks, and presence of other diseases that might affect the interpretation of neutrophil counts such as hematological diseases, malignancies, systemic lupus erythematosus etc.

### Study design

At enrollment, participants were administered to lung function tests according to the standards of the American Thoracic Society/European Respiratory Society^39^. A detailed questionnaire was given to collect information about symptoms, COPD exacerbation history, smoking status and basic demographics. Then, eligible patients received inhaled fluticasone propionate/salmeterol combination (500/50 ug, twice daily) for the next 24 weeks. A trained nurse instructed inhaler technique for each patient and taken 2 ml venous blood from them for genotyping. In this study, we mainly evaluated ICS therapeutic response in patients with different genotypes, which was defined as changes in FEV_1_ (i.e. FEV_1treatment_ – FEV_1baseline_) after 24 weeks treatment. All the clinical data were collected by the same physician, who was blinded to patients’ genotypes. In addition, an extra 8 ml blood was collected for neutrophil culture *in vitro* from parts of voluntary patients and was not a prerequisite for enrollment in our study.

A separate written informed consent was obtained from all participants. This study was approved by the Ethical Committee of Tongji Hospital, Tongji Medical College, Huazhong University of Science and Technology (IRB ID: 20120401), and was conducted according to the principles of the Declaration of Helsinki. Sample collection and all the experimental methods were performed in accordance with the relevant guidelines and regulations.

### DNA extraction and genotyping

All participants were genotyped for the rs37973 polymorphism. Genomic DNA was extracted from 2 ml peripheral EDTA-anticoagulated blood according to the manufacturer’s protocol (Blood Genomic DNA Purification Kit; Tiangen Biotech, Beijing, China). Polymerase chain reaction amplification was performed in a total volume of 12.5 μl, containing 6.25 μl of Taqman Universal PCR Master Mix (Applied Biosystems Inc., Foster City, CA, USA), 50 ng of extracted DNA and 300 nM of specific probes and primers (Assay ID: rs37973, AHUACTL; Applied Biosystems). Reaction conditions were as follows: 95 °C for 10 min, 45 cycles at 95 °C for 15 s and 60 °C for 1 min. Genotypes were then determined by the Allelic Discrimination Program using the ABI 7900 HT TaqMan sequence detection system (Applied Biosystems). And 10% of samples were randomly genotyped in technical duplicates.

### Neutrophil isolation and culture

Neutrophils were isolated from peripheral blood by hydroxyethyl starch sedimentation, Ficoll–Hypaque density gradient centrifugation (TBD, Tianjin, China) and hypotonic lysis of contaminating erythrocytes (Becton Dickinson, Franklin Lakes, NJ, USA). The isolated cells were washed twice and resuspended in RPMI 1640 medium (Life Technologies/GIBCO, Carlsbad, CA, USA) containing 10% fetal bovine serum at 1 × 10^5^ cells/ml. Neutrophil viability consistently exceeded 90%, as determined by trypan blue exclusion test. The cell suspension was then transferred equally to 6-well non-adherent culture plates (Corning, NY, USA), and divided into two groups: with or without 10 μg/ml cigarette smoke extract (Murty Pharmaceuticals, Inc., Lexington, KY, USA). Cells of both groups were stimulated with different concentrations of dexamethasone (Sigma) 0, 10^−6 ^M and 10^−4^ M in a solvent of dimethyl sulphoxide and were incubated in a humidified incubator (95% air, 5% CO_2_, 37 °C) for 18 hours. All experiments were performed in triplicate and repeated three times.

### Detection of apoptosis by flow cytometry

After incubation, the percentage of neutrophil apoptosis was quantitatively determined by Annexin V-FITC Apoptosis Detection Kit (KeyGen, Nanjing, China) according to the manufacturer’s protocol. In brief, cells were harvested, washed once with ice-cold phosphate-buffered saline and then resuspended in 400 μl Annexin V binding buffer. After incubating with 4 ul Annexin V-FITC (Ann V) for 10 min at room temperature in the dark, 4 ul propidium iodide (PI) were added and incubated for another 5 min. All the experimental procedures were strictly operated on ice. Samples were then immediately run on a Becton Dickinson LSR flow cytometer with data analyzed by CellQuest software. The normal viable cells were defined as Ann V^−^/PI^−^, early apoptotic cells Ann V^+^/PI^−^, late apoptotic or necrotic cells were Ann V^+^/PI^+^ as a result of membrane damage.

### Statistical analysis

Categorical variables in baseline characteristics were compared with Pearson chi-square tests and continuous variables with one-way ANOVA, Bonferroni post hoc tests. Hardy-Weinberg equilibrium was analyzed, using chi-square test to compare the observed genotype frequencies with those expected genotype frequencies. The effect of rs37973 variant on changes in lung function after 24 weeks of ICS treatment was assessed by general linear model without adjustment, because of the homogeneous baseline stratified by genotype. Linear regression was used to estimate the effect of per minor allele ‘G’ of rs37973 (coded as 0, 1, or 2) on changes in FEV_1_.

In addition to rs37973 genotype, stepwise multiple linear regression was performed as an exploratory analysis of factors that could affect ICS therapeutic response, since previous studies have shown that age, sex, baseline percentage of predicted FEV_1_ and smoking status affect changes in FEV_1_^19^,^20^. Smoking status was significant predictor of changes in FEV_1_ in our study, and then we repeated our analyses with an additional factorial analysis of covariance. Fixed effects were smoking status and rs37973 genotype, with three covariates: age, sex and baseline percentage of predicted FEV_1._

Analyses of dexamethasone-mediated neutrophil apoptosis were performed by two-tailed paired t-tests or independent-samples t-tests, when compared within or between the rs37973 genotype. Statistical analyses were performed using IBM SPSS Statistics 22.0 on Windows (SPSS Inc., Chicago, IL, USA) and *p* value less than 0.05 was considered statistically significant.

### Data Availability

This study was registered in the Chinese Clinical Trials Registry, http://www.chictr.org.cn/; registration number, ChiCTR-ROB-15005824. Date of registration, 2015-01-05.

## Additional Information

**How to cite this article**: Lei, Y. *et al*. *GLCCI1* rs37973: a potential genetic predictor of therapeutic response to inhaled corticosteroids in Chinese chronic obstructive pulmonary disease patients. *Sci. Rep.* **7**, 42552; doi: 10.1038/srep42552 (2017).

**Publisher's note:** Springer Nature remains neutral with regard to jurisdictional claims in published maps and institutional affiliations.

## Figures and Tables

**Table 1:** Demographic and baseline characteristics of patients according to the rs37973 genotype.

Characteristic	Total (N = 204)	AA	AG	GG	*p* value
		(N = 59)	(N = 95)	(N = 50)	
Male sex	162 (79.4)	42 (71.2)	79 (83.2)	41 (82.0)	0.177
Age (years)	67.0 ± 12.6	68.1 ± 11.8	66.7 ± 12.8	66.4 ± 13.2	0.739
Current smoker^†^	109 (53.4)	30 (50.8)	49 (51.6)	30 (60.0)	0.561
Pack-years smoked	26.6 ± 31.2	29.5 ± 37.2	24.7 ± 30.8	26.7 ± 23.8	0.643
GOLD stage at enrollment					0.125
30–50%: GOLD 3	71 (34.8)	17 (28.8)	41 (43.2)	13 (26.0)	
<30%: GOLD 4	48 (23.5)	12 (20.3)	21 (22.1)	15 (30.0)	
Other category^‡^	85 (41.7)	30 (50.8)	33 (34.7)	22 (44.0)	
FEV_1_ at baseline (L)	1.22 ± 0.59	1.29 ± 0.65	1.18 ± 0.56	1.20 ± 0.60	0.511
(% of predicted)	46.31 ± 20.06	50.69 ± 21.91	44.51 ± 18.57	44.56 ± 20.10	0.138

Data are presented as number (%) or mean ± standard deviation. *P* > 0.05 for all characteristics according to the One-Way ANOVA with a Bonferroni post hoc test or Pearson Chi-square Tests.

GOLD denotes Global Initiative for Chronic Obstructive Lung Disease. FEV_1_, forced expiratory volume in one second.

^†^Current smokers are patients smoking with more than 20 pack-years.

^‡^Patients in this category have a history of at least one chronic obstructive pulmonary disease exacerbation in the previous year before screening.

## References

[b1] Global, regional, and national age–sex specific all-cause and cause-specific mortality for 240 causes of death, 1990–2013: a systematic analysis for the Global Burden of Disease Study 2013. *The Lancet* **385**, 117–171 (2015). 10.1016/S0140-6736(14)61682-2 PMC4340604 25530442

[b2] Hooper R. . Risk factors for COPD spirometrically defined from the lower limit of normal in the BOLD project. Eur. Respir. J. 39, 1343–1353 (2012). 22183479 10.1183/09031936.00002711 PMC3378500

[b3] Vestbo J. . Global strategy for the diagnosis, management, and prevention of chronic obstructive pulmonary disease: GOLD executive summary. Am. J. Respir. Crit. Care Med. 187, 347–365 (2013). 22878278 10.1164/rccm.201204-0596PP

[b4] Calverley P. M. . Salmeterol and fluticasone propionate and survival in chronic obstructive pulmonary disease. N. Engl. J. Med. 356, 775–789 (2007). 17314337 10.1056/NEJMoa063070

[b5] Marzoratti L., Iannella H. A. & Waterer G. W. Inhaled corticosteroids and the increased risk of pneumonia. Ther. Adv. Respir. Dis. 7, 225–234 (2013). 23445751 10.1177/1753465813480550

[b6] Battaglia S., Cardillo I., Lavorini F., Spatafora M. & Scichilone N. Safety considerations of inhaled corticosteroids in the elderly. Drugs Aging 31, 787–796 (2014). 25212953 10.1007/s40266-014-0213-1

[b7] Izuhara Y. . GLCCI1 variant accelerates pulmonary function decline in patients with asthma receiving inhaled corticosteroids. Allergy 69, 668–673 (2014). 24673601 10.1111/all.12400

[b8] Hosking L. . GLCCI1 rs37973 does not influence treatment response to inhaled corticosteroids in white subjects with asthma. J. Allergy Clin. Immunol. 133, 587–589 (2014). 24131825 10.1016/j.jaci.2013.08.024 PMC3960323

[b9] Thompson B. . S31 Variation at GLCCI1: Association with Increased Steroid Dose But Not Adrenal Suppression in Asthmatic Children. Thorax 67, A17.12–A17 (2012).

[b10] McGeachie M. J. . Predicting inhaled corticosteroid response in asthma with two associated SNPs. Pharmacogenomics J. 13, 306–311 (2013). 22641026 10.1038/tpj.2012.15 PMC3434304

[b11] Hu C. . GLCCI1 Variation Is Associated with Asthma Susceptibility and Inhaled Corticosteroid Response in a Chinese Han Population. Arch. Med. Res. 47, 118–125 (2016). 27133712 10.1016/j.arcmed.2016.04.005

[b12] Tantisira K. G. . Genomewide association between GLCCI1 and response to glucocorticoid therapy in asthma. N. Engl. J. Med. 365, 1173–1183 (2011). 21991891 10.1056/NEJMoa0911353 PMC3667396

[b13] van den Berge M., Hiemstra P. S. & Postma D. S. Genetics of glucocorticoids in asthma. N. Engl. J. Med. 365, 2434-2435; author reply 2435–2436 (2011). 22187996 10.1056/NEJMc1112547

[b14] Hoenderdos K. & Condliffe A. The neutrophil in chronic obstructive pulmonary disease. Am. J. Respir. Cell Mol. Biol. 48, 531–539 (2013). 23328639 10.1165/rcmb.2012-0492TR

[b15] Marwick J. Glucocorticoid insensitivity as a future target of therapy for chronic obstructive pulmonary disease. Int. J. Chron. Obstruct. Pulmon. Dis. 297 (2010). 10.2147/copd.s7390 PMC2939685 20856829

[b16] Chapman M. S., Askew D. J., Kuscuoglu U. & Miesfeld R. L. Transcriptional control of steroid-regulated apoptosis in murine thymoma cells. Mol. Endocrinol. 10, 967–978 (1996). 8843413 10.1210/mend.10.8.8843413

[b17] Hizawa N. Pharmacogenetics of chronic obstructive pulmonary disease. Pharmacogenomics 14, 1215–1225 (2013). 23859575 10.2217/pgs.13.107

[b18] Kim W. J. . Association between CRHR1 polymorphism and improved lung function in response to inhaled corticosteroid in patients with COPD. Respirology 14, 260–263 (2009). 19210659 10.1111/j.1440-1843.2008.01425.x

[b19] Celli B. R. . Effect of pharmacotherapy on rate of decline of lung function in chronic obstructive pulmonary disease: results from the TORCH study. Am. J. Respir. Crit. Care Med. 178, 332–338 (2008). 18511702 10.1164/rccm.200712-1869OC

[b20] Scanlon P. D. . Smoking cessation and lung function in mild-to-moderate chronic obstructive pulmonary disease. The Lung Health Study. Am. J. Respir. Crit. Care Med. 161, 381–390 (2000). 10673175 10.1164/ajrccm.161.2.9901044

[b21] Marwick J. A. . Oxygen levels determine the ability of glucocorticoids to influence neutrophil survival in inflammatory environments. J. Leukoc. Biol. 94, 1285–1292 (2013). 23964116 10.1189/jlb.0912462 PMC3855024

[b22] Cox G. Glucocorticoid treatment inhibits apoptosis in human neutrophils. Separation of survival and activation outcomes. J. Immunol. 154, 4719–4725 (1995). 7722324

[b23] Zhang X., Moilanen E. & Kankaanranta H. Beclomethasone, budesonide and fluticasone propionate inhibit human neutrophil apoptosis. Eur. J. Pharmacol. 431, 365–371 (2001). 11730731 10.1016/s0014-2999(01)01437-6

[b24] GlaxoSmithKline. *PGx6951: Analysis of influence of GLCCI1 variant rs37973 on inhaled corticosteroid response in COPD patients treated with fluticasone furoate in* HZC112206 and HZC112207 https://www.gsk-clinicalstudyregister.com/study/200367#rs (2014).

[b25] Postma D. S., Kerkhof M., Boezen H. M. & Koppelman G. H. Asthma and chronic obstructive pulmonary disease: common genes, common environments? Am. J. Respir. Crit. Care Med. 183, 1588–1594 (2011). 21297068 10.1164/rccm.201011-1796PP

[b26] Hogg J. C. . The nature of small-airway obstruction in chronic obstructive pulmonary disease. N. Engl. J. Med. 350, 2645–2653 (2004). 15215480 10.1056/NEJMoa032158

[b27] Barnes P. J. Immunology of asthma and chronic obstructive pulmonary disease. Nat. Rev. Immunol. 8, 183–192 (2008). 18274560 10.1038/nri2254

[b28] Nittoh T. . Effects of glucocorticoids on apoptosis of infiltrated eosinophils and neutrophils in rats. Eur. J. Pharmacol. 354, 73–81 (1998). 9726633 10.1016/s0014-2999(98)00426-9

[b29] Meagher L. C., Cousin J. M., Seckl J. R. & Haslett C. Opposing effects of glucocorticoids on the rate of apoptosis in neutrophilic and eosinophilic granulocytes. J. Immunol. 156, 4422–4428 (1996). 8666816

[b30] Telenga E. D., Kerstjens H. A., Postma D. S., Ten Hacken N. H. & van den Berge M. Inhaled corticosteroids in chronic obstructive pulmonary disease: a review. Expert Opin. Pharmacother. 11, 405–421 (2010). 20102305 10.1517/14656560903510628

[b31] Bleecker E. R., Emmett A., Crater G., Knobil K. & Kalberg C. Lung function and symptom improvement with fluticasone propionate/salmeterol and ipratropium bromide/albuterol in COPD: response by beta-agonist reversibility. Pulm. Pharmacol. Ther. 21, 682−688 (2008). 18541448 10.1016/j.pupt.2008.04.003

[b32] Miravitlles M., Soler-Cataluna J. J., Calle M. & Soriano J. B. Treatment of COPD by clinical phenotypes: putting old evidence into clinical practice. Eur. Respir. J. 41, 1252–1256 (2013). 23060631 10.1183/09031936.00118912

[b33] Kitaguchi Y., Komatsu Y., Fujimoto K., Hanaoka M. & Kubo K. Sputum eosinophilia can predict responsiveness to inhaled corticosteroid treatment in patients with overlap syndrome of COPD and asthma. Int. J. Chron. Obstruct. Pulmon. Dis. 7, 283–289 (2012). 22589579 10.2147/COPD.S30651 PMC3346210

[b34] Anthonisen N. R., Connett J. E. & Murray R. P. Smoking and lung function of Lung Health Study participants after 11 years. Am. J. Respir. Crit. Care Med. 166, 675–679 (2002). 12204864 10.1164/rccm.2112096

[b35] Jen R., Rennard S. I. & Sin D. D. Effects of inhaled corticosteroids on airway inflammation in chronic obstructive pulmonary disease: a systematic review and meta-analysis. Int. J. Chron. Obstruct. Pulmon. Dis. 7, 587–595 (2012). 23055709 10.2147/COPD.S32765 PMC3459653

[b36] Lapperre T. S. . Effect of fluticasone with and without salmeterol on pulmonary outcomes in chronic obstructive pulmonary disease: a randomized trial. Ann. Intern. Med. 151, 517–527 (2009). 19841453 10.7326/0003-4819-151-8-200910200-00004

[b37] Holloway R. A. & Donnelly L. E. Immunopathogenesis of chronic obstructive pulmonary disease. Curr. Opin. Pulm. Med. 19, 95–102 (2013). 23325031 10.1097/MCP.0b013e32835cfff5

[b38] Cazzola M. & Dahl R. Inhaled combination therapy with long-acting beta 2-agonists and corticosteroids in stable COPD. Chest 126, 220–237 (2004). 15249466 10.1378/chest.126.1.220

[b39] Miller M. R. . Standardisation of spirometry. Eur. Respir. J. 26, 319–338 (2005). 16055882 10.1183/09031936.05.00034805

